# Tidal volumes: cold and dry or warm and humid, does it matter?

**DOI:** 10.1007/s10877-019-00416-7

**Published:** 2019-11-14

**Authors:** Mats Wallin, Göran Hedenstierna

**Affiliations:** 1 Department of Physiology and Pharmacology, Karolinska Institute, Stockholm, Sweden; 2 Maquet Critical Care AB, Röntgenvägen 2, 171 54 Solna, Sweden; 3 Department of Medical Sciences, Clinical Physiology, Uppsala University, Uppsala, Sweden

A patient connected to an ICU-ventilator without humidifier and with flawless Y-piece volume measurements who inspires 500 ml repeatedly expires about 560 ml. He, or she, will continue to have an intact lung function and not losing any lung volume. How come? The explanation is that in the lungs dry gas is humidified and heated to body temperature. The increase in temperature from 20 to 37 °C, which is equal to an increase from 293 to 310 K, expands the volume by 5.8% (310/293 = 1.058) according to Charles’s law (V~T). The rising temperature reflects an augmented kinetic energy of the molecules in the gas which expands the inherent volume. The gas is also humidified in the lung. Water molecules (vapour) are added until saturation. The number of molecules (n) in the initial dry gas is increased. From the descriptive universal gas law, PV = nRT, it can be seen that an increase of the number of molecules must be accompanied by an increase in either pressure (P) or volume (V). Water vapour saturation is temperature dependent and at 37 °C vapour saturation pressure is 47 mmHg which is 6.2% of the normal barometric pressure (760 mmHg). Accordingly, the volume expansion of an initial dry gas by vapour is about 6.2%. The net result from each contribution, temperature and humidity, is multiplicative, 1.058 × 1.062 = 1.123. Consequently, under a situation where we have no cuff leakage and a perfect volume measurement the expired tidal volume shall always be larger than the inspired one since the inspired gas is colder and dryer. This everyday occurrence is not always obvious. The reason is that manufacturers compensate for the increase in temperature and humidity and present a reduced expired volume according to the applied dry and cold reference condition, but also the generally limited accuracy in volume measurement, in both ICU-ventilators and anaesthesia machines contributes. Both factors conceal the gas expansion phenomenon within the lung.

The reasoning above elucidates why the conversion factor between the two common reference conditions used during tidal volume measurements, ATPD [ambient temperature (here 20 °C) and pressure, dry] and BTPS (body temperature ambient pressure, saturated), is 12%.

In previous anaesthesia machine and ICU-ventilator standards, manufacturers were allowed to freely choose which reference condition (ATPD, BTPS) to use. A device from a manufacturer using BTPS as the reference delivers a tidal volume from its outlet that is 12% smaller than the one delivered by a device based on ATPD when an ATPD calibrated flowmeter is used.

The volume expansion of a gas volume from heat and humidity described above is fairly easy to comprehend. Nevertheless, the conversion of gas volume measurements during different reference conditions must be considered complicated and it is very easy to make mistakes. If the conversion is based on wrong assumptions the result will inevitably be erroneous. An example that illustrates these difficulties is the study by Wallon et al. published in BJA 2013 [1]. When comparing the volume delivered by different anaesthesia machines these authors reported invalid conclusions because their calculations were based on incorrect assumptions.

However, in the latest anaesthesia machine and ventilator standards from 2011, it is stated that “gas volumes, flow rates, and leakage in the breathing circuit shall be expressed at BTPS” [2, 3]. The standards also state that dry gases shall be used during tests. Both requirements of the new standards make conversion between ATPD and BTPS a necessity in all manufacturers’ laboratories. The reason for the dry gas statement is that it is much easier to work with dry gases. Humidity is cumbersome to handle because condensation occurs when temperature decreases. Furthermore, ordinary flow meters and test lungs are normally more precise and accurate during dry conditions.

Do the new requirements in the standards have any practical clinical impact? Why were the standards changed when it is so much easier to work with dry and room tempered gas? The aim of this editorial is to clarify the benefits from the BTPS-requirement but also enlighten the principal clinical consequences of it.

An obvious benefit is that all ventilators and anaesthesia machines deliver and present volume and flow according to the same reference condition. By this alteration the 12% inborn volume uncertainty between ATPD- and BTPS-calibrated ventilators is eliminated. A given set tidal volume will cause the same lung expansion regardless of the manufacturer which facilitates ventilator comparative studies and eradicates one confounding factor [the reference condition (ATPD, BTPS) variability] in lung protective ventilation studies. (A set tidal volume of 6 ml/kg corresponds to a delivery from the ventilator of only 5.36 ml/kg if the ventilator refers to BTPS.)

As clinicians our mind-set is that we set the tidal volume we want the ventilator to deliver to the patient. This is also in line with the definition of delivered volume presented in the new standards which is “the volume entering the patient’s respiratory tract” [2, 3]. Most of us also have the apprehension that the volume reaching the patients’ lungs not differs from the volume delivered by the ventilator outlet, but this actually only applies when dry gases are used as in the test lab. In most clinical situations however, this is not the case.

**Fig. 1:**
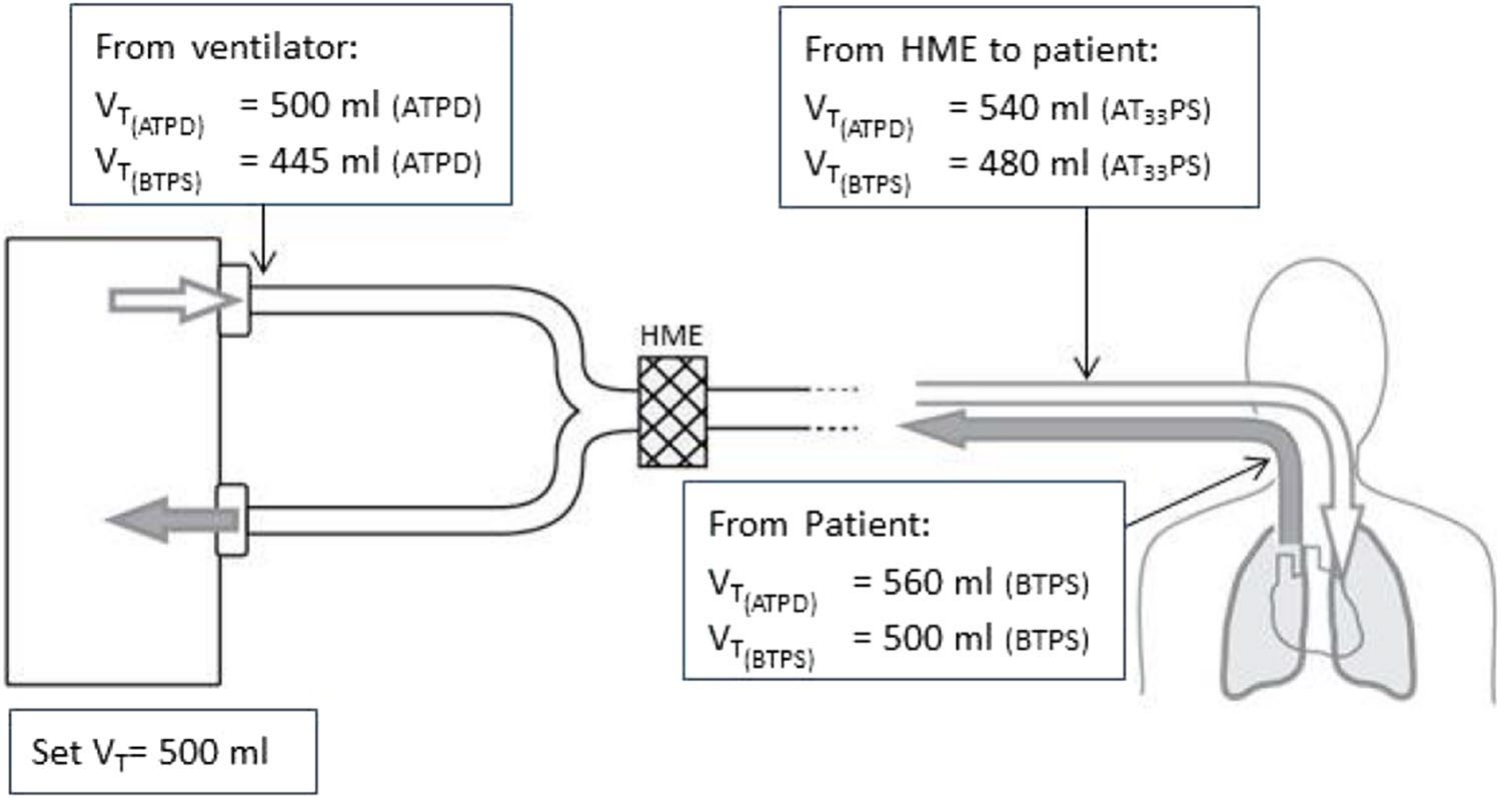
An ICU-ventilator supplied with compressed gases delivers dry gases from its outlet. The figure describes the volume expansion phenomena from heat and humidity and the volume delivery difference between an ATPD- and BTPS-calibrated ICU-ventilator. Set tidal volume (V_T_) is 500 ml. The gas volume entering the patient’s respiratory tract differs from set tidal volume when a HME is used in both cases. V_T(ATPD)_ tidal volume for a ventilator using ATPD as reference condition. Ambient temperature is in this example 20 °C, V_T(BTPS)_ tidal volume for a ventilator using BTPS as reference condition, *HME* heat and moisture exchanger, *AT*_*33*_*PS* ambient temperature 33 °C, saturated

In a non-rebreathing wall-gas dependent ICU-ventilator, the CO_2_ produced by the patient is eliminated by the fresh gas. A constant delivery of dry fresh gas will drain out the patient’s mucous membrane and consequently the gas must be humidified which is commonly performed by a passive humidifier, a heat moisture exchanger (HME), or sometimes by an active humidifier. A HME humidifies the inspired dry gas to a relative humidity of about 80% but the surrounding environment of the HME has a cooling effect and as a consequence the gas entering the tracheal tube has a temperature of about 33 °C [4]. Thus, in an ICU-ventilator a HME expands the dry gas coming out from the ventilator outlet by 7–8% so the actual gas volume entering the patient’s respiratory tract is 7–8% larger than the volume delivered at the ventilator outlet. However, inhaled gas volume is still 4–5% smaller than the corresponding exhaled volume which has body temperature and is saturated (BTPS). An ATPD-calibrated ventilator delivering 500 ml dry gas from the outlet supplies the lungs with about 540 ml due to humidification and rise in temperature by the HME whereas a for a BTPS calibrated ventilator the post-HME volume entering the tracheal tube is 480 ml when an ATPD calibrated spirometer is used. Both present V_Ti_ as 500 ml on the display. Within the patients’ lungs the inspired gas further expands and the patients’ lungs will be enlarged with 560 ml (in the ATPD ventilator) and 500 ml (in the BTPS ventilator) respectively (Fig. 1). The exhaled volume, when measured in the close proximity (no cooling) of the airway, corresponds to the enlargement of the lung. Both ventilators will however present the same exhaled volume (V_Te_), 500 ml, since the ATPD-ventilator converts and presents the volume at dry and ambient conditions (ATPD). Accordingly, the gas volume entering the patient’s respiratory tract differs in clinical practice from the volume delivered by the ventilator due to ordinary clinical humidification and heating. Only during true dry and ambient conditions “the volume entering the patient respiratory tract” will be the same as the volume delivered by the ventilator.

A similar reasoning as above can be done for anaesthesia machines but then gas temperature and humidity at the Y-piece is fresh gas flow dependent. The inspired gas temperature at the Y-piece in a circle system is commonly 26–28 °C during minimal flow anaesthesia without HME [5]. If a HME is used at the Y-piece the temperature may increase to 32–33 °C again depending on the fresh gas flow [5, 6]. Consequently, the actual tidal volume entering the patient’s respiratory tract varies up to about 10% in circle system anaesthesia just due to fresh gas flow dependent humidity and temperature. This is a regular clinical event which means that a tidal volume inaccuracy of 10% shall be accepted and not be seen as a clinical risk. As in ICU-ventilators the final expansion of the inspired gas to BTPS-condition occurs in the patient’s lungs.

As mentioned above volume measurements at a true BTPS condition (tempered 37 °C and saturated gas) are cumbersome due to the huge risk for condensation and the fact that most test lungs and flow meters are not commonly designed for humid applications. A technique for accurate measurements regardless humidity is to measure pressure and temperature in a “Tank lung”, filled with copper wool, in a heated climate chamber. The volume is measured (read calculated) by applying the universal gas law on the obtained pressure and temperature values.

To conclude, during anaesthesia gas temperature and humidity at the Y-piece is depending on the fresh gas flow used. Consequently, the tidal volume entering the patient’s respiratory tract can vary up to about 12% with unchanged volume settings. The temperature and humidity of the gas at the Y-piece will always be somewhat cooler and dryer than the gas within the lung. The body conveys extra energy to the incoming gas by adding water vapour and increasing gas temperature. The consequence will be a further volume expansion within the lung. A BTPS calibrated ventilator delivers a tidal volume representing the lung expansion. The exhaled volume, measured in close proximity to the airway, reflects this tidal lung expansion.

So does this new BTPS-requirement in the standards matter? For patients most likely not at all, but clinicians ought to change their mind-set to be correct. We now set how large the patient’s lung expansion shall be and not which volume the ventilator shall deliver.
